# Functional Effects of Parasites on Food Web Properties during the Spring Diatom Bloom in Lake Pavin: A Linear Inverse Modeling Analysis

**DOI:** 10.1371/journal.pone.0023273

**Published:** 2011-08-22

**Authors:** Boutheina Grami, Serena Rasconi, Nathalie Niquil, Marlène Jobard, Blanche Saint-Béat, Télesphore Sime-Ngando

**Affiliations:** 1 Université de La Rochelle-CNRS, UMR 6250 Littoral Environnement et Sociétés (LIENSs), La Rochelle, France; 2 LMGE, Laboratoire Microorganismes: Génome et Environnement, UMR CNRS 6023, Clermont Université, Blaise Pascal, BP 80026, Aubière, France; Argonne National Laboratory, United States of America

## Abstract

This study is the first assessment of the quantitative impact of parasitic chytrids on a planktonic food web. We used a carbon-based food web model of Lake Pavin (Massif Central, France) to investigate the effects of chytrids during the spring diatom bloom by developing models with and without chytrids. Linear inverse modelling procedures were employed to estimate undetermined flows in the lake. The Monte Carlo Markov chain linear inverse modelling procedure provided estimates of the ranges of model-derived fluxes. Model results support recent theories on the probable impact of parasites on food web function. In the lake, during spring, when ‘inedible’ algae (unexploited by planktonic herbivores) were the dominant primary producers, the epidemic growth of chytrids significantly reduced the sedimentation loss of algal carbon to the detritus pool through the production of grazer-exploitable zoospores. We also review some theories about the potential influence of parasites on ecological network properties and argue that parasitism contributes to longer carbon path lengths, higher levels of activity and specialization, and lower recycling. Considering the “structural asymmetry” hypothesis as a stabilizing pattern, chytrids should contribute to the stability of aquatic food webs.

## Introduction

Fungal parasitism is common in plankton communities [1]–[4], especially in the form of parasitic chytrids [5]–[8]. In freshwater environments, chytrids infect a wide variety of hosts including fish, eggs, zooplankton, and other aquatic fungi but primarily microalgae. Chytrids are also called ‘zoosporic’ fungi [9], since their life cycle includes dispersal forms, uniflagellated zoospores, and host-associated infective sporangia. Microscopic observations have provided evidence for the presence of both forms in the plankton [8], [10].

Previous studies have investigated the effects of chytrid parasitism on the growth of algal species and phytoplankton successions [11]–[13] as well as on the genetic structure of infected populations [14]. A critical finding was that chytrids seem to preferentially infect large algae. In the absence of parasites, large and colonial microalgae species because of their inedibility are unexploited in the planktonic food web and sink from the euphotic to the benthic zone [15], [13]. However, infective sporangia not only consume host biomass thereby contributing to nutrient recycling, but also produce zoospores which are potential prey for cladoceran zooplankter [16]. Thus, fungi might increase the growth of phytoplankton through releasing nutrients bound in inedible algae, and increase the growth of zooplankton by converting biomass from inedible algae to edible zoospores. Moreover, zoospores constitute upgraded food source for grazers because of their high nutritional quality [16]. This chytrid pathway was recently conceptualized in the “Mycoloop theory” [13], and in the specific case of large inedible algae, chytrid zoospores can constitute an important trophic link and prevent the loss of energy in the plankton. Thus, parasitic chytrids are potentially important in pelagic environments due to their role as trophic links and in biogeochemical cycling.

Given that food webs are central to ecological concepts [17], it is important to establish the role of parasites in the structure and function of food webs. In theory, parasites can have a variety of effects. Lafferty et al. [18]–[19] suggested that parasites affect food-web properties and topology since they double connectance (defined as the number of observed links divided by the number of possible links) and quadruple the number of links. Others have postulated that parasites drive an increase in species richness, trophic levels, and trophic chain length of the food web [20]–[21]. These properties may stabilize community structure [22]. However, the potential effects of parasites on food web stability is a complex and unresolved issue [19] since the concept of stability is the centre of a perhaps infinite debate in community ecology [23]–[28]. Based on the ideas of May [29], parasites should lead to a destabilized trophic network because they increase species diversity and the connectance. In addition, adding parasites to food webs extends the length of trophic chains which can decrease food-web stability [30]. However, the addition of long loops of weak interactions, which may be a characteristic of parasites with complex life cycles, might offset the destabilizing effects of increased connectance [31].

To investigate ecosystem properties and ecological theories, the application of mathematical tools, such as models, is useful and allows trophic network representation through carbon flows. In the absence of quantification of the flows induced by fungal activity, simulations were recently realized of their potential role in the plankton food web of the Lake Biwa [32]. The presence of this indirect pathway channeling microphytoplankton production to the consumers via the fungi, leads to an enhancement of the trophic efficiency index and a decrease of the ratio detritivory/herbivory [32], when considering the fungi, compared with a model without fungi. The results suggested that the food web relies less on the consumption of detritus, and that the transfer of carbon to higher trophic levels is higher than estimated without taking into account the parasites. Due to the lack of data quantifying carbon transfer through parasitism in pelagic ecosystems no attempt was made to build model based on field estimated flows. Thus, the roles and ecological implications of chytrid infections of microphytoplankton remain to be fully explored for aquatic microbial food webs.

In this study our objective was to add parasitic chytrids as a compartment in a well-studied pelagic food web and quantify their impact on matter flow through a trophic network. We then evaluate, for the first time, the impact of chytrid parasitism on the functioning of a planktonic ecosystem using field data set collected from the euphotic zone of the oligo-mesotrophic Lake Pavin (Massif Central, France). To describe the food web, we built a model representative of carbon flow involved in chytrid parasitism and quantified the amount of primary production channelled in food web via chytrid infection. Specifically, we compared carbon flows between the complete food web including parasitic chytrids (MWC, Model with Chytrids) with the same model that did not consider the presence of chytrids and the resulting flows (MWOC, Model without Chytrid), as traditionally done in previous plankton food-web analysis [e.g. 33]. Both of them were constructed on the base of the same data set corresponding to the spring bloom period in Lake Pavin (i.e. March to June 2007).

These models were built using the Linear Inverse Modeling procedure (LIM, [34]) recently modified into the LIM-Monte Carlo Markov Chain (LIM-MCMC, [35]). This method allow reconstruction of missing flow values and alleviates the problem of under-sampling using the principle of conservation of mass, i.e. the quantity of carbon coming into each compartment considered as equal to the amount leaving it [34]. Thanks to recent development of the inverse analysis into LIM – MCMC, a probability density function covering the range of possible values, was generated for each flow. For each calculated set of flows, there is a set of calculated indices which allows application of statistical tests. The flows obtained from the models were used for calculations of Ecological Network Analysis indices that characterize the structure of the food web, and help reveal emergent properties [36]–[38]. The use of ecological indices moreover, allows an indirect evaluation of the effects of network properties on the stability of an ecosystem, as several authors have proposed theoretical links between structural properties and local stability. For example, Ulanowicz [39] stated that a stable ecosystem needs sufficient amounts of two mutually exclusive attributes: ecosystem organization and overheads. Ecosystem organization is characterized by the Ascendency index [40]. Overheads represent all those disorganized, inefficient and incoherent aspects of an ecosystem's activity [41]. In the absence of perturbation, ecosystem Ascendency tends to increase at the expense of the overheads and the ultimate result is a highly organized, tightly constrained and stable ecosystem [39]. On the other hand, Rooney et al. [42] proposed as a stabilizing pattern a high specialisation of the flows (comparable to the high organisation proposed by Ulanowicz) in the lowest trophic levels and a low specialisation in the high levels. The sum of these analyses revealed overlooked trophic links in a planktonic system in which parasites of microalgae are integrated including (i) the carbon flows involved in chytrid parasitism pathway, (ii) the emergent properties of a planktonic food web in which parasites are integrated, and (iii) the structural and functional properties of a parasite-containing ecosystem.

## Results

### 1. Direct and indirect impacts of chytrids on overall flows

**Figure 1:**
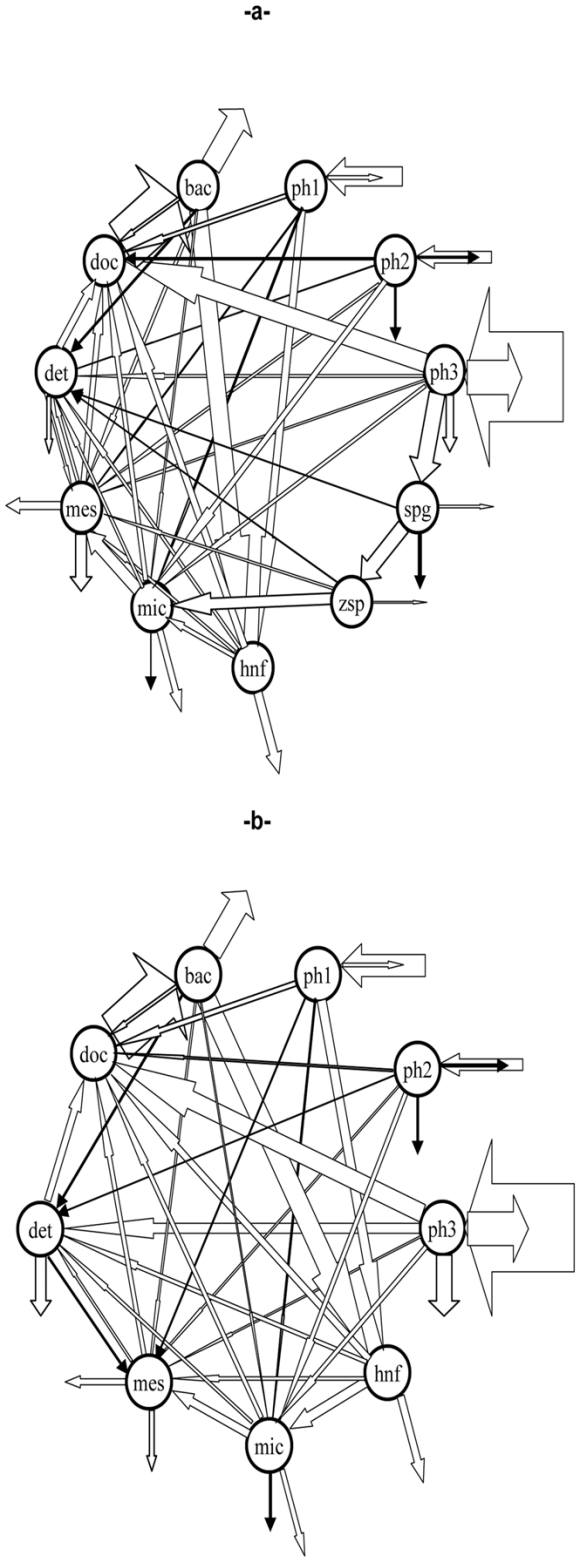
Food webs constructed by LIM-MCMC approach: a- Model With Chytrids and b- Model Without Chytrids. Footnotes: Used abbreviations: bac: heterotrophic bacteria; ph1, ph2 and ph3 are pico-, nano- and microphytoplankton compartments; hnf: heterotrophic nanoflagellates; mic and mes: micro- and mesozooplankton compartments; zsp: zoospores, spg: sporangia; det: detritus and doc: dissolved organic carbon. Green arrows indicate gross primary production, arrows pointing away from center of each compartment indicate respiration, and arrows pointing down represent loss by sedimentation flows. Widths of arrows indicate magnitude of carbon flow.

Calculated flows (mgCm^−2^ d^−1^) by the LIM–MCMC method, in food webs with or without fungal compartments (i.e. MWC and MWOC), are detailed in Fig. 1 and Table 1. Carbon input into the pelagic system was from the phytoplanktonic primary producers. No allochtonous input were considered due to the small catchment area (50 ha) of Lake Pavin, and the absence of inflow river. For the two models, the major contribution to the gross primary production was provided by microphytoplankton (74%) while the nanophytoplankton and the picophytoplankton contribution were at 16% and 10% respectively. The carbon throughput calculated for sporangia and zoospores represented 5.2% and 4.2% of the total system throughput, respectively.

**Table 1:** Flow description, name and corresponding value (mg C m^−2^ d^−1^) of steady state models of the pelagic food web of Lake Pavin during spring 2007.

		Inferred value (mg C m^−2^ d^−1^)	
		Model I	Model II
Flow description	Flow name	With Chytrid	Without Chytrid
Microphytoplankton gross primary production	CgppTOph3	267.8	261.63
Nanophytoplankton gross primary production	CgppTOph2	35.27	33.94
Picophytoplankton gross primary production	CgppTOph1	57.47	64.96
Microphytoplankton respiration	Cph3TOres	**101.67**	**100.49**
Microphytoplankton doc excretion	Cph3TOdoc	**56.11**	**55.57**
Microphytoplankton grazing by mic	Cph3TOmic	**11.01**	**14.86**
Microphytoplankton grazing by mes	Cph3TOmes	**6.13**	**5.94**
Parasitism of ph3 by sporangia	Cph3TOspg	57.44	
Microphytoplankton det production	Cph3TOdet	**9.36**	**29.75**
Microphytoplankton sinking	Cph3TOlos	26.07	55.03
Nanophytoplankton respiration	Cph2TOres	**2.53**	**4**
Nanophytoplankton doc excretion	Cph2TOdoc	**3.75**	**6.03**
Nanophytoplankton grazing by mic	Cph2TOmic	**18.24**	**14.77**
Nanophytoplankton grazing by mes	Cph2TOmes	**9.71**	**6.62**
Nanophytoplankton sinking	Cph2TOlos	**0.52**	**1.28**
Nanophytoplankton det production	Cph2TOdet	**0.51**	**1.25**
Picophytoplankton respiration	Cph1TOres	**11.16**	**10.88**
Picophytoplankton doc excretion	Cph1TOdoc	**13.2**	**16.41**
Picophytoplankton grazing by hnf	Cph1TOhnf	**25.85**	**31.98**
Picophytoplankton grazing by mic	Cph1TOmic	**5.1**	**4.18**
Picophytoplankton grazing by mes	Cph1TOmes	**2.16**	**1.51**
Bacteria respiration	CbacTOres	**73.27**	**71.75**
Bacterivory by hnf	CbacTOhnf	61.59	67.24
Bacteria uptake by mes	CbacTOmes	**10.87**	**6.88**
Bacteria uptake by mic	CbacTOmic	**5.98**	**4.09**
Bacterial doc release due to viruses lysis	CbacTOdoc	9.9	9.9
Attached bacteria to det	CbacTOdet	**1.67**	**1.88**
Heterotrophic nanoplankton respiration	ChnfTOres	**29.25**	**25.1**
Heterotrophic nanoplankton doc excretion	ChnfTOdoc	**23.1**	**21.91**
Heterotrophic nanoplankton uptake by mic	ChnfTOmic	**14.41**	**29.61**
Heterotrophic nanoplankton uptake by mes	ChnfTOmes	**10.27**	**10.07**
Heterotrophic nanoplankton det production	ChnfTOdet	**10.39**	**12.54**
Microzooplankton respiration	CmicTOres	**21.87**	**16.94**
Microzooplankton doc excretion	CmicTOdoc	**16.27**	**13.94**
Microzooplankton uptake by mes	CmicTOmes	**38.14**	**24.57**
Microzooplankton egestion	CmicTOdet	**10.44**	**9.66**
Microzooplankton sinking	CmicTOlos	1.38	2.4
Mesozooplankton respiration	CmesTOres	**24.16**	**18.29**
Mesozooplankton doc excretion	CmesTOdoc	**17.88**	**14.98**
Mesozooplankton egestion	CmesTOdet	**10.7**	**7.77**
Mesozooplankton grazing by larger organisms	CmesTOlos	**39.25**	**17.1**
Sporangia respiration	CspgTOres	**6.9**	
Sporangia emission of zoospores	CspgTOzsp	46.79	
Sporangia detrital production	CspgTOdet	**0.86**	
Sporangia sinking	CspgTOlos	**2.89**	
Zoospores respiration	CzspTOres	**5.92**	
Zoospores ingestion by mic	CzspTOmic	**33.34**	
Zoospores ingestion by mes	CzspTOmes	**6.63**	
Zoospores detrital production	CzspTOdet	**0.9**	
Dissolved organic carbon uptake by bacteria	CdocTObac	**163.27**	**161.75**
Detritus dissolution	CdetTOdoc	**23.06**	**23.01**
Detritus consumption by mes	CdetTOmes	8.07	2.55
Detritus sinking	CdetTOlos	**14**	**37.3**

Model II presents no fungi while model I consider them in the model exercise. Underlined values indicate flows that were estimated or derived from processes determined *in situ* in Lake Pavin. Bold values are those constrained by one or 2 inequations and estimated by the LIM-MCMC method.

**Figure 2:**
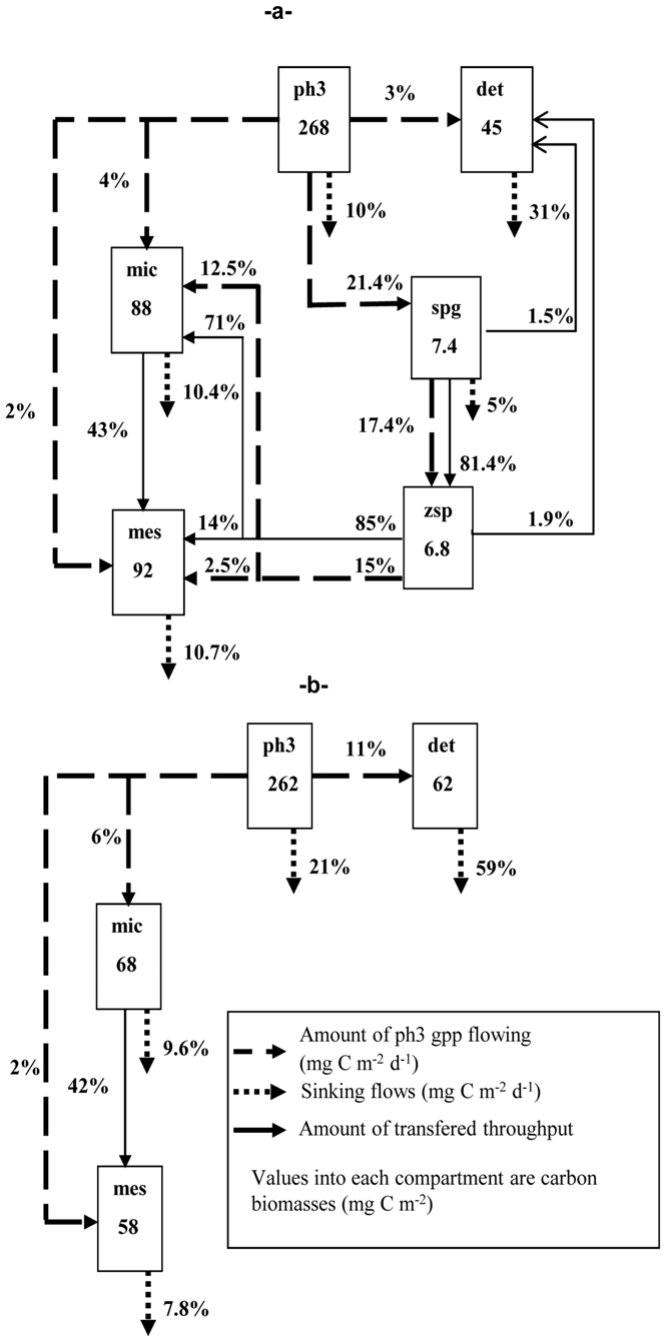
Highlights of the carbon sinking and flowing from ph3 gross primary production to other compartments for a- MWC, and b- MWOC.

Carbon flowing from the microphytoplankton compartments to the parasites (i.e. sporangia and zoospores) and through them to the grazers (i.e. microzooplankton and mesozooplankton) is detailed in Fig. 2 in order to focus and compare the fate of microphytoplankton primary production for both models. Through parasitism, 21.4% of the microphytoplankton throughput was channelled to sporangia compartment (Fig. 2); 17.4% of this flow was transferred to zoospores via sporangia production and 12.5% was then channelled to microzooplankton and 2.5% to mesozooplankton by grazing on zoospores. Among fungal compartments, 81.4% of sporangia throughput was channelled to zoospores and 85% of zoospores throughput was channelled to micro- and mesozooplankton. Most of this flow was taken up by microzooplankton (71%) and only 14% by mesozooplankton. In the presence of chytrids a substantial contribution to microzooplankton diet originated from zoospores (38% of total diet).

Formation of detritus represented 9% of total sporangia compartment throughput, the losses by respiration and sedimentation were at 12 and 5%, respectively. Detritus formation from zoospores constituted 1.8% of this compartment throughput, the carbon loss by respiration was 12.6% and no loss due to sinking was considered. The total detritus formation decreased in the presence of chytrids in the food web (29% less of detritus compartment throughput) due to lower contribution of microphytoplankton (from 11 to 3% ) to detritus input (Fig. 2).

Compared to the model without chytrids, microzooplankton and mesozooplankton throughput in the model with parasites exhibited higher throughput rates, 23% and 36%, respectively. Conversely, heterotrophic nanoflagellates and detritus throughputs increased (13% and 40%, respectively) when parasites were not included in the food web construction (i.e. MWOC).

**Figure 3:**
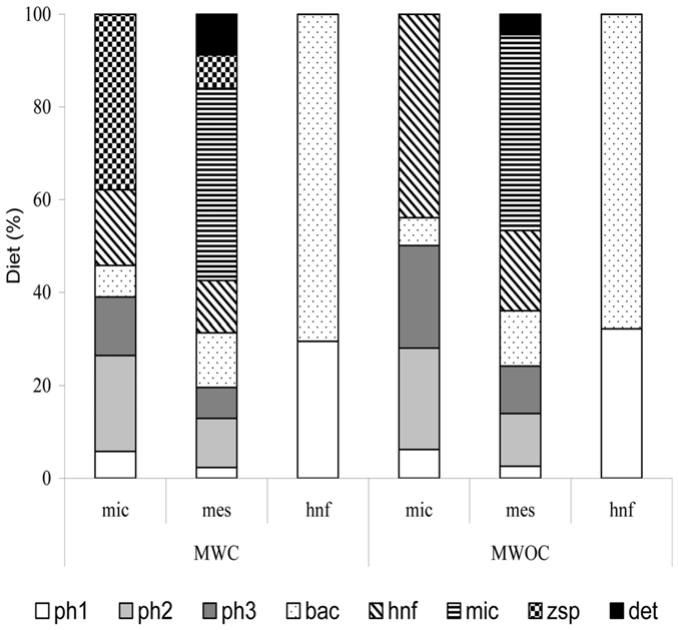
Diet composition of hnf, mic and mes in the two models.

Grazing insured direct transfer of 6% of microphytoplankton carbon produced to microzooplankton and 2% to mesozooplankton. As a consequence, the total input flow from microphytoplankton to grazers increased from 8% in the model without chytrids to 21% in the model with chytrids (Fig. 2). In the model without chytrids the most important contribution to microzooplankton diet was heterotrophic nanoflagellates (44%) (Fig. 3). Considering the mesozooplankton, the most important contribution was that of microzooplankton for both models (42% of total diet, Fig. 3) but the flow from micro- to mesozooplankton in term of mgC m^−2^ d^−1^ was higher in MWC (38 vs. 24 mgC m^−2^ d^−1^).

The loss by sinking flows was lower in the MWC than in the MWOC models due to higher detritus and microphytoplankton sinking flows in the second model. For microphytoplankton compartment the sinking flow was reduced from 21% to 10% of the compartment throughput in the MWC. In addition, due to less detritus originating from microphytoplankton in the MWC model, the amount of detritus sinking in the MWOC model was almost three times higher (Fig. 2).

### 2. Impact of chytrid on network emerging properties

**Figure 4:**
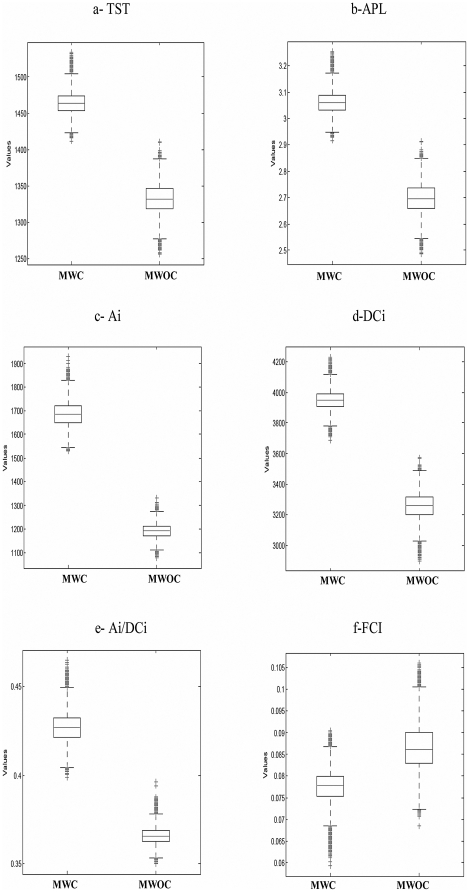
Comparison of Ecological Network Analysis indices across food webs with (MWC) and without (MWOC) Chytrids.

The LIM-MCMC derived flow results of each model were used to calculate the ecological network indices. Box plots of Fig. 4 show the relative positions of the median and associated dispersion of some ecological network analysis indices of the two models. For all considered indices, lower (0.5%) and upper (99.5%) quantiles did not overlap. It was straightforward to see differences in network properties of the two considered webs. Indeed, comparison (by Student test) of the same index in the two different models showed that the effect of chytrid inclusions was significant (p<0.05).

Chytrid increased the total activity, measured by total system throughput (TST, Fig. 4a) and the calculated averages for MWC and MWOC were respectively at 1464 and 1332 mgCm^−2^ d^−1^. In the presence of chytrids (Fig. 4b) the average pathway length (APL) also was higher. This means that trophic chains were longer and the calculated mean number of compartments through which each inflow passed was equal to 3 in the model with chytrid (MWC) and 2.6 in the model without chytrid (MWOC).

Higher values of Ascendency (A) were calculated when chytrid were considered in the model food web (2305 and 2812 mgC m^−2^ d^−1^ respectively for MWOC and MWC). Then, parasitism led to a trophic network with higher fraction of TST efficiently transferred along specialized pathways. The relative Ascendency (A/DC), a ratio reflecting the degree of organization [40], [43] was higher in the MWC (60%) compared to the MWOC (58%). Since A is calculated as the product of the TST and the average mutual information, any increase of A could be linked to an enhancement of TST with a concomitant decrease in the information factor (AMI). In this case we did observe an increase of the TST values for the MWC (Fig. 4a). Furthermore, the calculated AMI showed higher values for the MWC (1.92) compared to the MWOC (1.73). Indices relative to internal exchanges (Ai and DCi) showed higher values calculated for the MWC (Fig. 4c and 4d). Indeed, higher Ai/DCi ratio reported for the MWC demonstrated its tendency to internalize most of its activity which could be an aspect of a highly organized food web [43]. In contrast, the absence of chytrids promote enhancing the internal relative redundancy (MWC: 57%; MWOC: 63%) a measure of the ecosystem degree of information loss due to parallel pathways. Also, chytrids decrease recycling as indicated by a decrease of the Finn Cycling Index (FCI, Fig. 4f).

The Lindeman spine provided more insights considering trophic analysis. According to the trophic level efficiencies and to the global trophic efficiency given in Table 2, the most efficient planktonic food web for carbon transfer was the MWC. Moreover, the grazing chain analysis highlighted a better efficiency transfer of the primary production in the MWC for each trophic level (Table 2). The food web percentage of detritivory was higher in the MWOC (67%) compared to the MWC (55%). Detritivory decrease when considering chytrids in the spring pelagic food web of Lake Pavin.

**Table 2:** Indices derived from the Lindeman spine.

	Model I -With Chytrid	Model II -Without Chytrid
Trophic level (TL) efficiency (%)		
1^st^ TL	66.4	53.9
2^nd^ TL	48.0	42.3
3^rd^ TL	41.9	30.8
4^th^ TL	30.5	22.9
Global trophic efficiency	44.9	35.6
Grazing chain efficiency (%)		
1^st^ TL	37.7	22.1
2^nd^ TL	40.7	28.5
3^rd^ TL	17.1	8.8
4^th^ TL	5.2	2.0
Detritivory (%)	55.7	67.3
Herbivory (%)	44.3	32.7

## Discussion

### 1. Methodological choices

Using Linear Inverse Modeling coupled with the Monte Carlo Markov Chain approach applied to a complete *in situ* data set on chytrids allowed construction of the first model that quantifies and describes the implications of fungal parasitism in a pelagic ecosystem. Ecological Network Analysis (ENA), increasingly used coupled with a calculated set of flows driven from modelling exercises [33], [44], allows characterisation of emergent properties and overcomes the difficulties of comparing different food webs solely on the basis of the magnitude of flows [45].

Monte Carlo approach is a technique only recently applied to the Linear Inverse Modeling (LIM) [46] to describe the whole set of possible solutions and to overcome the problem of adopting the parsimony principle [34] that was used traditionally and consistently underestimates both the size and the complexity of the food web [46]–[48]. The Monte Carlo Markov Chain technique was recently coupled with a new mirror algorithm (LIM-MCMC, Markov Chain Monte Carlo algorithm) to provide more robust ecological basis to the models with a high range of possible solutions [49]. Kones et al. [49] also proposed the first coupling of the LIM-MCMC with ENA indices calculation, which allows estimating a probability density function covering the whole space of possible solutions for each ENA index, for each model. This adds to the great quality of our data set concerning the parasites and the planktonic processes [50], as it allows comparing the ecological properties of the two food web properties (i.e. with and without parasites) with a statistical test. In the present paper, we indeed present the first application of this approach to an ecological question. Performing statistical tests on the calculated ENA indices has proved to be useful for our purpose to compare the classical plankton food web carbon model of the Lake Pavin with the model including the two life forms of chytrids (zoospores and sporangia).

### 2. Carbon flows involved in parasitism

Channeling of primary production through the food web (Fig. 2) was highly impacted by the inclusion of chytrids in the plankton ecosystem of Lake Pavin. About 21% of microphytoplankton primary gross production is involved in sporangia development and increased carbon input into the zooplanktonic compartments (i.e. micro- and mesozooplankton). In the classical food web vision with no parasites, biogenic carbon produced by phytoplankton was considered to pass through various processes including remineralisation, food wed transfer and loss by sinking. The most effective way to pass energy to consumers is via direct consumption processes, even through microbial loop pathways, i.e. DOC uptake by bacteria [51], and zooplankton grazing [52]. Another part of primary production is considered as lost by sinking without being consumed [53]. However, this study showed the importance of parasitism as an indirect pathway channelling primary produced carbon to grazers.

Grazers were directly sustained by parasitic chytrids through the consumption of their zoospores (38% of microzooplankton diet). This pathway, called ‘Mycoloop’ by Kagami *et al.* [13] was quantified in our study and shown to increase energy transfer from primary producers to consumers in the system. In the temperate Lake Pavin during spring period large-size diatoms sustain primary production (Fig. 1) and the principal species are the pennate genera *Asterionella* and *Synedra* and the filamentous genera *Melosira*. These large diatoms are usually too large to be eaten and thus are not considered as efficient links in a classical food web (Fig. 2b). Our model, including parasitism in the pelagic food web confirms and provides, for the first time, quantitative estimates of primary production contained in “inedible algae” but channeled into zooplankton (mainly microzooplankton) via the ‘Mycoloop’.

Chytrids zoospores do constitute a food source of high quality; they are rich in polyunsaturated fatty acids (PUFAs) and cholesterol, which are essential nutrients for crustaceans [16]. Moreover, it is also possible that grazers due to the absence of a chitinous cell wall could easily assimilate zoospores. In Lake Pavin, as in other lake environments [54], [55], fungal epidemics occur when diatoms form blooms during spring period. This is a crucial moment in seasonal development of planktonic successions [56], especially for crustacean zooplankton development. It has been shown that diatoms constitute the basic fuel for higher trophic levels at the start of the growing season [57]. Experimental evidence suggests that chytrids improve survival of Cladocera [16] and Copepods [57]. Kagami *et al.* [53] remarked that cell lysis due to fungal infection of the diatom species *Staurastrum dorsidentiferum* in Lake Biwa could lead to nutrient release, supporting the production of bacteria and zooplankton, but the mechanism involved remained unclear. It is possible that, at this crucial moment for planktonic community development, grazing on chytrid zoospores might facilitate the growth of zooplankton due to their high nutritional quality.

In our model, carbon flows increased when chytrids were included and carbon transfer to higher trophic levels was improved (Table 1, Fig. 2). This pathway appears to be extremely efficient, the flow to detritus from the compartment of sporangia represented only 9% of its total throughput and it was even less for zoospores (2%). Consequently, the carbon input that passed into the zooplankton (i.e. micro- and mesozooplankton) was higher in the model with chytrids (Fig. 2) than without. Fungal zoospores represent key intermediates in food chains [58] and a very efficient link between sporangia and grazers. This could be due to the fact that they constitute more direct producer-consumer link [58]. Zoospores do not feed [58], [59] and the losses by respiration are extremely low compared to equivalent compartments such as heterotrophic nanoflagellates, HNF (Fig. 1, Table 1).

The inclusion of parasitic chytrid drastically reduced the direct loss via sedimentation of large phytoplankton (50% less) and their role in the production of detritus (29% less, Fig. 1). It is known that diatoms are usually considered to sink fast due to the weight of the frustule. Especially, the species *Melosira* is known to proliferate in Lake Pavin [60], [61] and was observed reaching the hypolimnion when 40–60% of the cells were still viable [62]; thus the large diatoms could not constitute an efficient trophic link. It would therefore appear that in the absence of parasites the majority of algal production is lost by sinking and is unavailable to support higher trophic levels. The consequence of parasitism on microphytoplankton will be that less carbon is lost from the pelagic zone. The fate of sinking organic matter will depend on the studied lake. In the studied case, a meromictic lake, the sinking organic matter constitutes an energy supply for the anoxic benthic community and will not reach the upper layers by recirculation. In a well-mixed lake, the sinking organic matter constitutes an energy supply for benthos, and can reach the upper layers by recirculation only after being processed in benthic food webs. Thus, chytrid infections can increase the residence time of algal biomass in water column [50] and decrease the contribution of organic matter to the benthic community.Our results show that the “Mycoloop” reduces the sinking flows of inedible algae and the carbon produced by these latter is transferred to consumers via zoospores of chytrid parasites. Chytrids not only have important role in the fate of primary production, but also in the trophic upgrading necessary to boost the seasonal development of planktonic populations in a pelagic ecosystem.

### 3. Emergent properties of ecosystem functioning

Theoretical studies [18]–[19] have concluded that emergent properties of an ecosystem can be affected by parasites. Analyzing food web models with, and without, parasites was suggested by parasitologists as a means to confirm theories [21], [63]. In our case, the comparison between the two models, including the range of possible values for ENA indices, clarifies the effects of parasites on ecosystem properties.

Number of flows increased in the model with chytrids (Fig. 1, Table 1) as well as the total system activity (TST, Fig. 4a). Parasites are known to add links and species to food webs [19] observed in broad variety of environments, including terrestrial [64], estuarine [18], marine [21] and freshwaters [55]. Even if this seems logical, as for the addition of any species to a food web, this is especially true for parasites that have a free-living stage that can interact with other compartment and that may be eaten, as the chytrids zoospores.

Including parasites in food web increases the number of compartments through which each inflow passes and the APL (Fig. 4b) expressed higher chain length for the MWC. This important structural property (i.e. APL) defines the number of links in food web and energy transfer through its components [65]. Increasing the number of links increases the robustness of interactions [19] of an ecosystem, a measure of its overall organization [18]. Robustness indeed is considered as a parameter linked to the stability of the system [18], and according to this theory, incorporating parasitic chytrids in pelagic food webs increases stability. According to Williams and Martinez [30], any increase in the length of trophic chains should decrease food web stability.

Parasites help the food web to reach a higher limit of development, as revealed by the DCi values. The increase of the relative Ascendency as well as the internal relative Ascendency (Ai/DCi) (Fig. 4e) point to a higher level of organization following the theory developed by Ulanowicz [36], [37].The action of chytrids allowed a higher fraction of the total system throughput to pass along specialized pathways as revealed by the Ascendency values. However, to conclude on the effect of chytrids on the degree of specialization, the AMI value provided better information about the flow specialization. Indeed, this index was higher when considering the presence of chytrids in the spring food web model. This means that inclusion of parasites leads to a higher specialization corresponding to a more direct link (i.e. zoospores>microzooplankton) compared to the classical microbial chain (i.e. DOC>bacteria>HFN>microzooplankton). In a food web simulation including chytrids [32], an increase in relative ascendency was related to an observed increase in trophic efficiency. Relative to the classical microbial chain, the flows estimated showed a higher transfer of primary produced organic matter via parasitism and zoospores grazing. Major ascendency could be the consequence from this more direct link between producers and consumers. As the degree of organization and robustness of the interactions among species into a food web impacts the degree of stability [19], the presence of chytrids could be considered as a factor enhancing stability. Robustness corresponds to a measure of the quantity of perturbation that an ecosystem can support before changing to another state [66]. The higher the robustness, the more stable the food web is. Niquil *et al.* [32] suggested after simulating the probable impact of chytrids in Lake Biwa, that high relative ascendency calculated for the low trophic levels associated with lower specialisation at higher levels (i.e. structural asymmetry reported by Rooney *et al.* [42]) should confer stability to the food web. In this study, higher ascendency was apparent for the lower trophic levels; and if the compartments were based on the level of species, incorporating the specificity of chytrids should enhance the ascendency even more than what we have observed in this study.

The inclusion of chytrids enhances the overheads of the ecosystem under study, especially the internal redundancy. The difference between 1 and the relative Ascendency estimates the overhead of the system. Overheads represent all those disorganized, inefficient and incoherent aspects of an ecosystem's activity [41]. In fact, they correspond to inputs, outputs, dissipation (respiration) and functional redundancy [67]. The functional redundancy can be estimated by the difference between Internal Ascendency and internal capacity of development. A higher functional redundancy in the presence of chytrids would enhance the stability. Indeed, as proposed by Ulanowicz [68], organized (Ascendency) and non-organized (redundancy) parts of ecosystem should be in equilibrium. This configuration of ecosystem leads to a better resistance and survival to perturbations. In the case of perturbations, an ecosystem with a high Ascendency would be less vulnerable and the consequences of perturbations would be small. The coupling with a high redundancy enhances such flexibility, and the dissipation of the effects of flow fluctuation as well [69].

The FCI index was lower in the model with chytrids. FCI is a measure of the quantity of recycling in the ecosystem and its low level confirms the tendency of parasites to favour the more direct links. In terrestrial environments, a theory elaborated for macroparasites [70] stated that parasites lead to complex long-loops interactions between species. Neutel *et al.* [31] stated that the addition of long loops of weak interactions could be the characteristic of parasites with complex life cycles. Here, the parasites studied had no significant effects on the recycling, because they only interacted with primary producers. One may consider that adding the parasites of heterotrophs would have had a different effect.

The calculated values of global trophic efficiency showed that carbon is rather efficiently transferred within the MWC. Reasons behind this high trophic efficiency are a better carbon transfer efficiency coupled with a higher grazing efficiency at each trophic level (Table 2). The MWC exhibited also a lower percentage of detritivory compared to the MWOC (Table 2), stressing the fact that in the MWC the food web is more efficient in direct use of the primary production compared with the MWOC food web. This conclusion is congruent with the conclusion made on the basis of carbon flows analysis. Moreover, detritivory decrease when parasites were involved could be related to the seasonal planktonic successions. Spring period in temperate lakes is a typical transitional system characterized by recovery of planktonic populations. Primary production is high and, in general, the community is dominated by r-strategist populations characterized by rapid growth and an important role of detritus in nutrient turnover [71]. This functional characteristic of an ecosystem is typical of immature community, as described in a previous study where during spring period detritivory value was high [72]. Including parasites was associated with a decrease in detritus consumption, enhancing the detritivory and thereby the maturity of the system. This fact related to low retention of the system (i.e. low FCI) contradicts the Odum [72] theory which states that efficient cycling of matter is a fundamental characteristic of a mature system.

### 4. Conclusions

This study is the first attempt to quantify the effective impact of parasitic chytrids in a pelagic food web. The improved inverse method coupled with Ecological Network Analysis allowed estimation of undetermined flows, taking into account the quantified processes from fungal infections and the emergent properties of the ecosystem. Methodological improvements allowed definition of the allowed range of values thus permitting statistical tests.

The epidemic growth of chytrids reduced the flow of sedimentation loss of large inedible algae and their contribution to detritus pool, thus less carbon is lost from the pelagic zone. Chytrid zoospores, via the Mycoloop pathway, channelled up to 20% of primary production to the higher trophic levels. The better global trophic efficiency was highlighted by a higher global trophic efficiency coupled to higher efficiency of carbon transfer through each trophic level of the grazing chain. Chytrids constituted an efficient and good quality trophic link and had important role in the flow of energy for the seasonal development of planktonic successions.

Parasitism affected also the structural and functional properties of the ecosystem. It lead to an increase in the number of the trophic links leading to longer path lengths, which in general are considered as characteristics of stable ecosystems. The increase in topological indices (i.e. A/DC, Ai/Dci and AMI) indeed indicated that parasites contribute to better organization of the ecosystem and more efficient specialization of pathways corresponding to links that are more direct. Some structural properties (i.e. FCI, detritivory) were indicative of a system based on rapid growth and turnover, thus of planktonic successions not yet mature and typical of spring period, and parasite seems to accentuate these characteristics.

## Materials and Methods

### 1. Study site and sampling

Lake Pavin is a crater freshwater lake situated in the Massif Central of France (45° 29′ 41″N, 02° 53′ 12″E). It is an oligo-mesotrophic deep volcanic mountain lake (Z_max_ = 92 m) with a permanently anoxic monimolimnion from 60 m depth downwards. More details on the characteristics of the lake are available in Amblard *et al.* [73]. The data used here correspond to the recurrent spring bloom of diatom in Lake Pavin, a site that offers a unique environment with low human influences and high annual reproducibility of seasonal dynamics in the water column [5]–[6]. Samples were collected fortnightly in a central location of the lake from March to June 2007 by simple capillarity as described by Sime-Ngando and Hartmann [74]. This method allowed collecting integrated samples (21 L) representative of the euphotic layer (0–20 m). Samples were pre-filtered on 150 µm pore size nylon filter (except for metazooplankton samples) for the elimination of metazoan zooplankton and taken to the laboratory for immediate analysis.

### 2. Abundance and biomass of planktonic organisms

Sub-samples were processed for identification and quantification of picoplankton, heterotrophic nanoflagellates, phytoplankton, zooplankton and the two life stages of microphytoplankton fungal parasites (Chytridiales).

#### Bacteria

Sample aliquots were fixed with glutaraldehyde (1% final concentration) before counting of heterotrophic and autotrophic picoplankton by a flow cytometer (BD system) equipped with a 15-mW, 488 nm, and air-cooled argon ion laser. Environmental samples were diluted 10 times and simultaneous measurements of size light scatter (relative size), 90 degree light scatter, and SYBR green fluorescence emission (wave length 650 nm) were conducted to detect and enumerate heterotrophic and autotrophic bacteria. Cell numbers were converted to carbon biomass by adopting mean cell volume (µm^3^). Carbon conversion factors of 0.35 pg C µm^−3^ and 0.22 pg C µm^−3^ were used for conversion of biovolumes to carbon biomasses of heterotrophic bacteria [75] and picophytoplankton (0.2–2 µm) [76], [77], respectively.

#### Heterotrophic nanoflagellates (2–20 µm)

Sub-samples (15 ml) were fixed and handled according to Caron [78] for quantification of heterotrophic nanoflagellates. Counts were performed using an inverted epifluorescent microscope (Leica DMIRB) equipped with a 100× objective lens, a HBO-100 W mercury lamp, and a set of different optic filters, including filters (340 to 380 nm) for UV light excitation. Mean cell biovolumes were estimated for each sample by measuring the linear dimension of at least 50 cells and equating shapes to standard geometric forms. Carbon biomass was calculated using a conversion factor of 0.22 pg C µm^−3^ [79].

#### Nano- and microphytoplankton (2–20 µm and 20–150 µm, respectively)

Sub-samples (200 ml) were fixed with alkaline Lugol solution (1% v/v) and nano- and microphytoplankton cells counted and identified using the Utermöhl method [80] under an inverted microscope (WILD - M40). Cell biovolumes were estimated by measuring the linear dimension of at least 100 cells and equating shapes to standard geometric forms. The resulting volumes were transformed into organic carbon values by using the conversion equation of Menden-Deuer and Lessard [81] (pgC cell^−1^ = 0.288×Vol^(0,811)^ for diatoms and pgC cell^−1^ = 0.216×Vol^(0,939)^ for the other autotrophic genera).

#### Ciliates (20–150 µm)

Sub-samples (200 ml) were fixed with alkaline Lugol solution (5% v/V) and ciliates were counted and identified using the same method as for microphytoplankton. For carbon biomasses of ciliates, biovolumes were converted into organic carbon using conversion factors of 0.19 pg C µm^−3^ [82].

#### Metazooplankton

The metazooplankton was collected by filtering raw samples from the euphotic layer (0–20 m) through a 50 µm pore-size mesh. Retained animals were preserved in 4% formalin-sucrose to prevent any release of eggs or physical deformation [83]. Identification and counting, after addition of few drops of rose Bengal to improve detection, were conducted under a binocular microscope (Wild M3Z) using Dolfuss chambers [84]. The carbon biomass of each metazoan group was estimated by multiplying the individual carbon contents by the corresponding abundances. For Copepods the dry weight (DW, mg) was calculated as 22.5% of wet weight [85], [86] and C content (mg) was estimated as 40% of DW [87]. For Cladocera the length (L, mm) of each organism was used to determine its carbon content (C_clad_) as: µg C ind^−1^ = 5.24×L−1.08 [88]. For rotifers, wet weights were converted to dry weight according to Pace and Orcutt [89] and McCauley [90]. Dry weights were converted to carbon biomass using carbon: DW ratio of 0.48 [91].

#### Chytrid parasites

Sub-samples were handled for chytrid parasites counting based on a size fraction approach and the use of the fluorochrome calcofluor white (CFW) for diagnosing, staining and counting chitinaceous fungal parasites (i.e. sporangia of chytrids) of microphytoplankton. 20 L of the integrated samples were passed through a 25 µm pore size nylon filter. Large phytoplankton cells in the >25 µm size fraction were collected and fixed with formaldehyde (2% final conc.), before staining and analysis. Nanoplanktonic cells in the <25 µm size-fraction were concentrated by ultrafiltration and 180 ml of the ultrafiltrate retentate was fixed with formaldehyde (2% final conc.), before staining and analysis. Aliquots (150 µl) of each fraction were stained by CFW (1% v/v) and drops (10 µl) of stained samples were mounted between glass slides and cover slips for observation and counting under an inverted epifluorescent microscope (more details are available in [8]). For each sample, microphytoplanktonic cells were inspected for fungal infection (i.e. the presence of fixed sporangia). Identification of chytrids was based on phenotypic keys known from classical manuals, primarily those in Sparrow [9], Canter [92], and Canter and Lund [93]. The prevalence of infection was estimated as the percentage of infection in the host population according to Bush *et al.* [94], i.e. Pr (%) = [(Ni/N)×100], where Ni is the number of infected host cells, and N is the total number of host cells. Carbon biomass of infectious sporangia was estimated using a conversion factor of 10.7 pg C cell^−1^ [13].

For zoospore counting, sub-samples were processed using the CARD-FISH method of Not *et al.* [95], recently modified by Jobard *et al.* [10]. Carbon biomass of zoospores was estimated using a conversion factor of 10.7 pg C cell^−1^ [13].

### 3. Model construction

Data from the field study were used to construct the pelagic food web model that quantitatively illustrates carbon pathways in Lake Pavin during spring in the presence of chytrids (sporangia and zoospore life stages). To estimate the unknown flows, we adopted the method LIM-MCMC, derived from the LIM of Vezina and Platt [34] to reconstruct trophic flows through the pelagic food web. The approach is based on four steps.

#### Compartments and a priori model

The first step consists in constructing a conceptual model including all possible flows between compartments and the outside. Living compartments included three phytoplankton compartments, three grazer compartments, one compartment for heterotrophic bacteria and two compartments for fungal parasites of microphytoplankton, while non-living compartments included dissolved organic carbon (doc) and detritus (det). We divided the phytoplankton into picophytoplankton (ph1: 0.2–2 µm); nanophytoplankton (ph2: 2–20 µm; principally Cryptophyta as *Rhodomonas sp.* and Chlorophyta as *Ankistrodesmus sp.* and *Ankyra sp.*) and microphytoplankton (ph3: 20–150 µm; essentially large and filamentous Bacillariophyceae, as *Asterionella sp.*, *Synedra sp.* and *Melosira sp.*). Grazer compartments included the heterotrophic nanoflagellates (hnf: 2–20 µm), microzooplankton (mic: 20–150 µm: Ciliates and some Rotifera: *Ascomorpha sp.*, *Keratella quadrata*, *Polyarthra platyptera* and *Conochilus unicornis*) and mesozooplankton (mes; >150 µm; Cladocera: *Bosmina coregoni*, *Daphnia longispina*; Copepoda: *Acanthodiaptomus denticornis*, *Cyclops abyssorum prealpinus* and some large Rotifera: *Brachionus sp.*, *Fillinia terminalis*, *Kellicottia longispina*). Fungal parasites of microphytoplankton compartments included infectious sporangia (spg) and free zoospores (zsp).

The food-web model contains 53 carbon flows, which correspond to the model with Chytrids (MWC). Since our aim was to highlight the role of chytrids in the pelagic food web and their impact on ecosystem properties, a second model was built with the same consideration and data set of the MWC but without incorporating the chytrids compartments and the involved flows. The second model had 44 carbon flows and was named model without chytrids (MWOC). The sole carbon inputs were gross primary productions by each phytoplankton size fraction. Carbon output from the network was driven by respiration of all living compartment and carbon loss by sinking from ph2, ph3, spg, mic, mes and det compartments. Mesozooplankton contribution to the carbon output flow considers not only their production of sinking fecal pellets but also their consumption by a higher trophic level. All living compartments, except fungal parasites, contribute to DOC production that was taken up by bacteria. In addition to ph2, ph3, mic and mes contributions to detritus production, we considered the existence of a carbon flow from bacteria and heterotrophic nanoflagellates to detritus. Attached bacteria were identified on TEP in Lake Pavin during spring [96] which sediment and create a flow from bacteria to detritus. Attached bacteria are also known to constitute preferential prey for heterotrophic nanoflagellates [97]. Detritus production by sporangia was due to chitinaeous wall dissolution or break-up during zoospore discharge [9]. Moreover, zoospores were considered as contributing to detritus production by the loss of their flagellum when they found a host to fix on. The carbon flow from microphytoplankton to sporangia represented carbon from diatom cells to infectious sporangia and the carbon flow from sporangia to zoospores was considered as the production of zoospores by sporangia. Grazing relationships were defined considering size and preferential ingestion of each identified grazer. Heterotrophic flagellates grazed on compartments of bac and ph1, microzooplankton grazed on bac, ph1, ph2, ph3, hnf and zsp, while mesozooplankton grazed on bac, ph2, ph3, hnf, mic, zsp and det.

#### Equalities

The second step is setting equations (equalities) to constrain the mass balance of the system and to impose measured flows. The mass balance equations for all compartments are given in the first 11 lines of Table 3. Some of the estimated flows were measured during previous studies focused on spring blooms in Lake Pavin and are introduced as additional equations; these include values for total gross and net primary production [98], [99], bacterial production [99] and viral lysis of bacteria [100] considered as the value of the flux from bacteria to DOC (last 4 lines of Table 3).

**Table 3:** Mass balance (1 to 11) and other linear (12 to 16) equations used for inverse analysis.

Equation number	Process concerned	Equations
1	Mass balance for microphytoplankton	(gpp-ph3)−(ph3-mic+ph3-mes+ph3-spg+ph3-doc+ph3-res+ph3-det+ph3-los) = 0
2	Mass balance for nanophytoplankton	(gpp-ph2)−(ph2-mic+ph2-mes+ph2-doc+ph2-res+ph2-det+ph2-los) = 0
3	Mass balance for picophytoplankton	(gpp-ph1)−(ph1-hnf+ph1-doc+ph1-res) = 0
4	Mass balance for heterotrophic nanoflagellates	(ph-hnf+bac-hnf)−(hnf-res+hnf-doc+hnf-mic+hnf-mes+hnf-det) = 0
5	Mass balance for bacteria	(doc-bac)−(bac-res+bac-doc+bac-hnf+bac-mic+bac-mec+bac-det) = 0
6	Mass balance for microzooplankton	(ph2-mic+ph3-mic+bac-mic+hnf-mic+zsp-mic)−(mic-mes+mic-doc+mic-res+mic-det+mic-los) = 0
7	Mass balance for mesozooplankton	(ph2-mes+ph3-mes+hnf-mes+mic-mes+det-mes+zsp-mes+bac-mes)−(mes-res+mes-doc+mes-det+mes-los) = 0
8	Mass balance for sporangia	(ph3-spg)−(spg-res+spg-zsp+spg-det) = 0
9	Mass balance for zoospores	(spg-zsp)−(zsp-res+zsp-mic+zsp-mes+zsp-det) = 0
10	Mass balance for detritus	(ph1-det+ph2-det+ph3-det+mic-det+mes-det+ext-det)−(det-doc+det-mic+det-mes+det-los) = 0
11	Mass balance for dissolved organic carbon	(ph1-doc+ph2-doc+ph3-doc+mic-doc+mes-doc+det-doc)−(doc-bac) = 0
12	Total gross primary production estimate	gpp-ph1+gpp-ph2+gpp-ph3 = 360.54*
13	Total net primary production estimate	(gpp-ph1+gpp-ph2+gpp-ph3)−(ph1-res+ph2-res+ph3-res) = 245.17*
14	Net bacterial production	doc-bac−bac-res = 90*
15	Viral lysis of bacteria	bac-doc = 9.90*

*values are in mgC m^−2^ d^−1^.

Primary production was measured from ^14^C uptake according to Steemann-Nielsen [101]. Subsamples (100 ml) from each sampling depth were drawn into two transparent and one dark (control) bottles, inoculated each with 0.5 µCi of NaH1 4C03 , and incubated in situ for 4 h. After incubation, 30 ml of each subsamples were filtered onto 1 µm pore size Nuclepore filter and the radioactivity was estimated by liquid scintillation counting. Calculation of total primary production and excreted primary production for each depth are given in details in [98], [99]. The value of net primary production was obtained after subtracting the value of the excreted primary production from the total primary production. Values corresponding for each depth were integrated to be representative of the 20 m water layer. Bacterial production was determined by measuring the uptake of tritiated thymidine into bacterial DNA [102], after incubating the samples for 45 min. The radioactivity was measured using a Beckman LS 5000 scintillation counter. The quantity of ^3^H-thymidine incorporated into DNA was converted into bacterial production by using a conversion factor of 2×10^18^ cells produced per mole of thymidine incorporated. The bacterial production was converted in terms of cells into the equivalent amount of carbon produced using the equation of Simon and Azam [103]. Viral lysis of bacteria was considered as the value of the flux from bacteria to DOC. In formalin-fixed samples, bacteria contained in 5 ml subsamples were harvested by ultracentrifugation onto three grids for 30 min at 4°C. Each grid was then stained for 30 s with uranyl acetate and examined with a transmission electron microscope. Bacterial cells containing mature phages were identified and counted. To estimate the impact of viruses on bacterial mortality, the used method proposed by Weinbauer et al. [104] was considered, based on transmission electron microscopy examinations of entire cells visibly infected by viruses. The fraction of bacterial mortality from viral lysis was related to the calculated frequency of visibly infected cells, calculations are detailed in Bettarel et al. [100].

#### Constraints

The third step consists in imposing ecological limits (maximum and/or minimum) for each unknown flow, which means a linear system of inequalities: 75 inequalities were provided for MWC and 68 inequalities for MWOC (fungi inequalities were not needed). These latter are presented, explained and referenced in Table S1. The first set of inequalities concerned the gross primary production partitioning between the three size fractions (ph1, ph2, ph3). Earlier works reported the higher contribution of diatoms (ph3) during spring to the total primary production compared to the pico- and the nanophytoplankton in Lake Pavin [62]. The maximum and the minimum contribution of each phytoplankton size class to the phytoplankton total biomass during spring 2007 in Lake Pavin were considered as upper and lower bounds of their contribution to the total gross primary production. Upper bound for chytrids respiration was considered as 20% of their total carbon uptake. In fact, sporangia are living fixed on their hosts from which they absorb their substrate [9], implying low energy loss from sporangia. Zoospore produced by sporangia have their own resource storage (carbohydrates, proteins, fatty acids, phospholipids, sterols and other lipids and nucleic acids; [105]) which is used for swimming away and to subsist until colonizing new substrates or infect new hosts [9]. To constrain the ingestion rate flows, preferential ingestion constraints were built based on taxa dominance, grazers and predators' cell or individual size and their corresponding prey size sampled during spring 2007. Bacterivory by heterotrophic nanoflagellates was constrained by a minimum value given by Bettarel *et al.* [100] for Lake Pavin. Ingestion rates of zoospores by microzooplankton were considered at least the double of those ingested by mesozooplankton. The lowest bound of carbon transfer from sporangia to zoospores was considered equal to zoospores biomass estimated for spring 2007.

#### Solutions

The last step of the inverse analysis is the calculation of flow solutions. A range of possible values was given by the adopted method of the Monte Carlo Markov Chain (LIM-MCMC), based on the mirror technique defined by Van Den Meersche *et al.* [35]. The jump value was set at 10 and a number of iterations of 100,000 was applied that proved optimal coverage of the possible solutions. The program used was a Matlab^©^ translation by Alain Vézina and Lauriane Campo of the R-CRAN project package LIM-Solve [35], [104]. More details on the method are available in Van Den Meersche *et al.* [35] and Niquil *et al.* [45].

### 4. Ecological Network Analysis

The resulting flows from inverse analysis were used for calculating indices from Ecological Network Analysis. We used an algorithm written for Matlab© by Carole Lebreton and Markus Schartau to calculate a set of indices that are used for describing the emergent properties of the ecosystem [36].

#### Total system throughput (TST)

TST is a measure of the total system activity and is the sum of all the flows through all compartments [97].

#### Average path length (APL)

APL is the average number of compartments crossed by a unit of carbon from its entry to the system to its living. It represents a measure of the system retention capacity [106].

#### System Ascendency (A)

System Ascendency (A) merges the quantification of the activity and the degree of specialization involved into processes [107]. It is the product of the TST and the average mutual information (AMI: degree of specialization of flows in the network) [36]. This value is a more informative metric describing the organization of the system when it is expressed in relation to development capacity that as its maximum value (A/DC). High A/DC ratios are in fact, thought to reflect high degrees of organization [43], [108]. The difference between A and its upper bound (DC) represents the overhead [109], which reflects the multiplicity of pathways in the network that happen for any of four reasons: uncertainty on imports (Oi), exports (Oe), dissipations (Do) and redundancy (R) [41]. The relative redundancy (R/DC, %) is a measure of the ecosystem degree of information loss due to parallel pathways.

#### Development capacity (DC)

Development capacity (DC) is calculated as the product of TST and the upper limit of AMI, corresponding to the maximum potential ascendancy and to a food web with maximum specialization. The development capacity is the sum of ascendency, redundancy and information loss related to external exchanges.

As suggested by Ulanowicz [36], growth and development were characterized by indices calculated over only internal exchanges. The internal capacity of ecosystems development (DCi) was calculated as the sum of internal ascendency (Ai) and internal redundancy (Ri). Any decrease of Ai/DCi ratio in relation to the A/DC ratio could point to a strong dependency of an ecosystem on external inputs [110]. Ai/DCi ratio could also be another aspect of a highly organized ecosystem with its tendency to internalize most of its activity [43]. However, Rutledge et al. [111] and others [108], [112] considered the internal redundancy (Ri/DCi) as a measure of ecosystem stability. Indeed, broken pathways are more easily re-established when the ecosystem exhibit higher internal stability or resilience to perturbation [43]. Resilience is the speed with which a system returns to equilibrium state after a perturbation [113], [114].

#### Finn Cycling Index (FCI)

FCI is the ratio of carbon flowing in loops to the sum of all carbon flows. It quantifies the fraction of all flows involved in recycling [115] and can be considered as a measure of the retentiveness of the system.

Trophic analysis: based on the trophic concept of Lindeman [116], maps the complex network of trophic transfers as a linear food chain (called Lindeman spine, [117]). The Lindeman spine allows calculation of the trophic efficiency for each level, i.e. the efficiency of transfer from one level to the next [107]. The global trophic efficiency is computed as the logarithmic mean of all the trophic level efficiencies. Two others indices were derived from the Lindeman spine the grazing chain efficiency and the percentage of detritivory. The mean values of the 100.000 set of flows resulting from the LIM-MCMC analysis were analyzed using the ecological network analysis package of Ulanowicz and Kay [118] to build the Lindeman spine. The program used for these analyses is available at www.glerl.noaa.gov/EcoNetwrk/.

### 5. Data analysis

Box plots, a widely used graphical tool introduced by Tukey [119], illustrate the location of the median and associated dispersion of the data. Box plots of all solutions per index for the model with chytrids were plotted with the model without chytrids. This method is useful as it provides visual information showing the differences in network properties of the two food-web models. The statistical comparison between the two constructed models was tested with two-tailed Student (t) tests on the sets of calculated ecological network indices.

## Supporting Information

Table S1 Constraints used on different planktonic food web processes.
(DOC)

## References

[1] Huber-Pestalozzi G (1946). Der Walensee und sein Plankton. In Canter, H. M. and Lund, J. W., 1948. Studies on plankton parasites. I. Fluctuations in the numbers of *Asterionella Formosa* Hass. In relation to fungal epidemics.. New phytol.

[2] Canter HM, Lund JWG (1969). The parasitism of planktonic desmids by Fungi.. Osterr Botan Zeitschrift.

[3] Sigee DC (2005). Freshwater microbiology..

[4] Tsui CKM, Hyde KD (2003). Freshwater mycology. Fungal diversity research series 10..

[5] Lefèvre E, Bardot C, Noël C, Carrias J.-F, Viscogliosi E (2007). Unveiling fungal zooflagellates as members of freshwater picoeukaryotes: evidence from a molecular diversity study in a deep meromictic lake.. Environ Microbiol.

[6] Lefèvre E, Roussel B, Amblard C, Sime-Ngando T (2008). The molecular diversity of freshwater picoeukaryotes reveals high occurrence of putative parasitoids in the plankton.. PloS One.

[7] Lepère C, Masquelier S, Mangot JF, Debroas D, Domaizon I (2010). Vertical structure of small eukaryotes in three lakes that differ by their trophic status: a quantitative approach.. ISME.

[8] Rasconi S, Jobard M, Jouve L, Sime-Ngando T (2009). Use of Calcofluor White for Detection, Identification, and Quantification of Phytoplanktonic Fungal Parasites.. Appl Environ Microbiol.

[9] Sparrow FK (1960). Aquatic Phycomycetes..

[10] Jobard M, Rasconi S, Sime-Ngando T (2010). Fluorescence in situ hybridization of zoosporic fungi (chytrids) in aquatic environments: testing with clone-FISH and application to natural samples using CARD-FISH.. J Microbiol Meth.

[11] Van Donk E, Sommer U (1989). The role of fungal parasites in phytoplankton succession,. Plankton Ecology: Succession in Plankton Communities.

[12] Ibelings BW, de Bruin A, Kagami M, Rijkeboer M, Brehm M (2004). Host parasite interactions between freshwater phytoplankton and chytrid fungi (Chytridiomycota).. J Phycol.

[13] Kagami M, de Bruin A, Ibelings BW, van Donk E (2007). Parasitic chytrids: their effects on phytoplankton communities and food-web dynamics.. Hydrobiologia.

[14] De Bruin A, Ibelings BW, Kagami M, Mooij WM, van Donk E (2008). Adaptation of the fungal parasite *Zygorhizidium planktonicum* during 200 generations of growth on homogeneous and heterogeneous populations of its host, the diatom *Asterionella formosa*.. J Euk Microb.

[15] Legendre L, Le Fèvre J, Demers S (1991). From individual plankton cells to pelagic marine ecosystems and to global biogeochemical cycles.. Particle analysis in Oceanography.

[16] Kagami M, von Elert E, Ibelings BW, de Bruin A, Van Donk E (2007). The parasitic chytrid, *Zygorhizidium* facilitates the growth of the cladoceran zooplankter, *Daphnia* in cultures of the inedible algae *Asterionella*.. Proceed Royal Soc Biol Sci.

[17] Pascual M, Dunne J (2006). Ecological Networks: Linking structure to dynamics in Food webs..

[18] Lafferty KD, Hechinger RF, Shaw JC, Whitney K, Kuris AM, Collinge S, Ray C (2006). Food webs and parasites in salt marsh ecosystem. Chap. 9,. Disease ecology: community structure and pathogen dynamics.

[19] Lafferty KD, Allesina S, Arim M, Briggs CJ, Leo G (2008). Parasites in food webs: the ultimate missing links.. Ecol Lett.

[20] Huxham M, Raffaelli D, Pike A (1995). Parasites and Food Web Patterns.. J Anim Ecol.

[21] Thompson RM, Mouritsen KN, Poulin R (2005). Importance of parasites and their life cycle characteristics in determining the structure of a large marine food web.. J Anim Ecol.

[22] Hudson PJ, Dobson AP, Lafferty KD (2006). Is a healthy ecosystem one that is rich in parasites?. Trends Ecol Evol.

[23] Elton CS (1958). The Ecology of Invasions by Animals and Plants..

[24] May RM (1972). Will a large complex system be stable?. Nature.

[25] Pimm SL (1984). The complexity and stability of ecosystems.. Nature.

[26] McCann KS (2000). The diversity-stability debate.. Nature.

[27] O'Gorman EJ, Emmerson MC (2009). Perturbations to trophic interactions and the stability of complex food webs.. Proceed Nat Acad Sci USA.

[28] Hosack GR, Li HW, Rossignol PA (2009). Sensitivity of system stability to model structure.. Ecol Model.

[29] May RM (1973). Stability and complexity in model ecosystems.

[30] Williams R, Martinez ND (2004). Simple rules yield complex food webs.. Nature.

[31] Neutel AM, Heesterbeek JAP, de Ruiter P (2002). Stability in real food webs: weak links in long loops.. Science.

[32] Niquil N, Kagami M, Urabe J, Christaki U, Viscogliosi E (2011). Potential role of fungi in plankton food web functioning and stability: a simulation analysis based on Lake Biwa inverse model.. Hydrobiologia.

[33] Niquil N, Bartoli G, Urabe J, Jackson GA, Legendre L (2006). Carbon steady state model of the planktonic food web of Lake Biwa, Japan.. Freshwater Biol.

[34] Vézina AF, Platt T (1988). Food web dynamics in the ocean. I. Best-estimates of flow networks using inverse methods.. Mar Ecol Prog Ser.

[35] Van den Meersche K, Soetaert K, Van Oevelen D (2009). xsample(): An R Function for Sampling Linear Inverse Problems.. J Stat Soft.

[36] Ulanowicz RE (1986). Growth and Development: Ecosystems Phenomenology..

[37] Ulanowicz RE (1997). Ecology, the Ascendent Perspective..

[38] Ulanowicz RE, Goerner SJ, Lietaer B, Gomez R (2009). Quantifying sustainability: resilience, efficiency and the return to of information theory.. Ecol complex.

[39] Ulanowicz RE (2003). Some steps toward a central theory of ecosystem dynamics.. Comput Biol Chem.

[40] Ulanowicz RE (1980). An hypothesis on the development of natural communities.. J Theor Biol.

[41] Ulanowicz RE (1988). On the importance of higher-level models in ecology.. Ecol Model.

[42] Rooney N, McCann K, Gellner G, Moore JC (2006). Structural asymmetry and the stability of diverse food webs.. Nature.

[43] Baird D, Mc Glade JM, Ulanowicz RE (1991). The comparative ecology f six marine ecosytems.. Phill Tran Roy Soc London Ser B.

[44] Grami B, Niquil N, Sakka Hlaili A, Gosselin M, Hamel D (2008). The plankton food web of the Bizerte Lagoon (South-Western Mediterranean): II. Carbon steady state modeling using inverse analysis.. Estuar Coast Shelf S.

[45] Niquil N, Soetaert K, Johnson GA, van Oevelen D, Bacher C, Wolanski E, Mc Lusky DS (2012). Inverse modelling in modern ecology and application to coastal ecosystem. Chap. 9 in Vol. 9 Estuarine and coastal ecosystem modelling (D. Baird & A. Mehta Editors). Treatise on coastal and estuarine Science.

[46] Kones JK, Soetaert K, van Oevelen D, Owino JO, Mavuti K (2006). Gaining insight into food webs reconstructed by the inverse method.. J Marine Syst.

[47] Niquil N, Jackson GA, Legendre L, Delesalle B (1998). Inverse model analysis of the planktonic food web of Takapoto Atoll (French Polynesia).. Mar Ecol Prog Ser.

[48] Johnson GA, Niquil N, Asmus H, Bacher C, Asmus R (2009). The effects of aggregation on the performance of the inverse method and indicators of network analysis.. Ecol Model.

[49] Kones JK, Soetaert K, Van Oevelen D, Owino JO (2009). Are network indices robust indicators of food web functioning? A Monte Carlo Approach.. Ecol model.

[50] Rasconi S (2010). Etude des chytrides parasites du phytoplancton dans les écosystèmes aquatiques..

[51] Azam F, Fenchel T, Field JG, Gray JS, Meyer-Reil LA (1983). The Ecological Role of Water-Column Microbes in the sea.. Mar Ecol Prog Ser.

[52] Yoshimizu C, Yoshida T, Nakanishi M, Urabe J (2001). Effects of zooplankton on the sinking flux of organic carbon in Lake Biwa.. Limnology.

[53] Kagami M, Gurung TB, Yoshida T, Urabe J (2006). To sink or to be lysed? Contrasting fate of two large phytoplankton species in Lake Biwa.. Limnol Oceanogr.

[54] Van Donk E, Ringelberg J (1983). The effect of fungal parasitism on the succession of diatoms in Lake Maarsseveen-I (The Netherlands).. Freshwater Biol.

[55] Kudoh S, Takahashi M (1990). Fundal control of population changes of planktonic diatom Asterionella Formosa in shallow eutrophic lake.. J Phycol.

[56] Sommer U, Gliwicz ZM, Lampert W, Duncan A (1986). PEG-model of Seasonal Succession of Planktonic Events in Fresh Waters.. Arch Hydrobiol.

[57] Kagami M, Helmsing NR, van Donk E (2010). Parasitic chytrids could promote copepod survival by mediating material transfer from inedible diatoms.. Hydrobiologia.

[58] Gleason FH, Kagami M, Marano AV, Sime-Ngando T (2009). Inoculum.. Newsletter Mycol Soc Amer.

[59] Canter HM, Jaworski GHM (1979). The Occurrence of a Hypersensitive Reaction in the Planktonic Diatom Asterionella formosa Hassall Parasitized by the Chytrid *Rhizophydium planktonicum* Canter Emend., in Culture.. New Phytol.

[60] Devaux J (1976). Dynamique des populations phytoplanctoniques dans deux lacs du massif central français.. Ann Sta Biol Besse-en-Chandesse.

[61] Amblard C (1978). Application du dosage des adenosines 5′-phosphate à l'étude d'un phytoplancton lacustre (Lac Pavin)..

[62] Amblard C, Bourdier G (1990). The spring bloom of the diatom *Melosira italica* subsp. subarctica in Lake Pavin: biochemical, energetic and metabolic aspects during sedimentation.. J Plank Res.

[63] Arias-Gonzalez JE, Morand S (2006). Trophic functioning with parasites: a new insight for ecosystem analysis.. Mar Ecol Prog Ser.

[64] Kuris AM, Hechinger RF, Shaw JC, Whitney KL, Aguirre-Macedo L (2008). Ecosystem energetic implications of parasite and free-living biomass in three estuaries.. Nature.

[65] Jordán F, Sheuring I, Molnár I (2003). Persistence and flow reliability in simple food webs.. Ecol Model.

[66] Loreau M, Downing A, Emmerson M, Gonzalez A, Hughes J, Loreau M, Naeem S, Inchausti P (2002). A new look at the relationship between diversity and stability.,. Biodiversity and ecosystem functionning.

[67] Baird D, Ulanowicz RE (1989). The Seasonal Dynamics of The Chesapeake Bay Ecosystem.. Ecol Monographs.

[68] Ulanowicz RE, Goerner SJ, Lietaer B, Gomez R (2009). Quantifying sustainability: Resilience, efficiency and the return of information theory.. Ecol Complexity.

[69] MacArthur R (1955). Fluctuations of Animal Populations and a Measure of Community Stability.. Ecology.

[70] Dobson AP, Lafferty K, Kuris A, Dunne JA, Pascual M (2005). Parasites and food webs.. Ecological Networks: Linking Structure to Dynamics in Food webs.

[71] Odum EP (1969). The strategy of ecosystem development.. Science.

[72] Marquis E, Niquil N, Delmas D, Hartmann HJ, Bonnet D (2007). Inverse analysis of the planktonic food web dynamics related to phytoplankton bloom development on the continental shelf of the Bay of Biscay, French coast.. Estuar Coast Shelf S.

[73] Amblard C, Rachiq S, Bourdier G (1992). Photolithotrophy, photoheterotrophy and chemoheterotrophy during spring phytoplankton development (Lake Pavin).. Microbial Ecol.

[74] Sime-Ngando T, Hartmann HJ (1991). Short-term variations of the abundance and biomass of planktonic ciliates in a eutrophic lake.. Eur J Protistol.

[75] Bjørnsen PK (1986). Automatic determination of bacterioplankton biomass by image analysis.. Appl Environ Microb.

[76] Søndergaard M, Jensen LM, Ertebrjerg G (1991). Picoalgae in Danish coastal waters during summer stratification.. Mar Ecol Prog Ser.

[77] Mullin MM, Sloan PR, Eppley RW (1966). Relationship between carbon content, cell volume and area in phytoplankton.. Limnol Oceanogr.

[78] Caron DA (1983). A technique for the enumeration of photosynthetic and heterotrophic nanoplankton using epifluorescence microscopy, and a comparison with other procedures.. Appl Environ Microb.

[79] Børsheim KY, Bratbak G (1987). Cell volume to cell carbon conversion factors for a bacterivorous *Monas sp.* enriched from seawater.. Mar Ecol Prog Ser.

[80] Utermöhl H (1931). Neue wege in der quantitativen erfassung des planktons (mit besonderer berücktingung des ultraplanktons).. Verh Int Ver Theor Angew Limnol.

[81] Menden-Deuer S, Lessard EJ (2000). Carbon volume relationships for dinoflagellates, diatoms and other protest plankton.. Limnol Oceanogr.

[82] Putt M, Stoecker DK (1989). An experimentally determined carbon: volume ratio for marine oligotrichous ciliates from estuarine and coastal waters.. Limnol Oceanogr.

[83] Prepas E (1978). Sugar-frosted Daphnia: an improved fixation technique for Cladocera.. Limnol Oceanogr.

[84] Dussart B, Boubée N, Cie (1967). Les Copépodes des eaux continentales d'Europe occidentale.. Calanoides et Harpactoides.

[85] Riemann F, Ernst W, Ernst R (1990). Acetate uptake from ambient water by the free-living marine nematode Adoncholaimus thalassophygas.. Mar Biol.

[86] Gradinger R, Friedrich C, Spindler M (1999). Abundance, Biomass and composition of the sea ice biota of the Greenland Sea pack ice.. Deep-Sea Res PT II.

[87] Feller RJ, Warwick RM, Higgings RP, Thiel H (1988). Energetics.. Introduction to the study of meiofauna.

[88] Kankaala P, Johansson S (1986). The influence of individual variation on length-biomass regressions in three crustacean zooplankton species.. J Plank Res.

[89] Pace ML, Orcutt D (1981). The relative importance of protozoans, rotifers, and crustaceans in a freshwater zooplankton community.. Limnol Oceanogr.

[90] McCauley E, Downing JA, Rigler FH (1984). The estimation of the abundance and biomass of zooplankton in samples.. A Manual for the Assessment of Secondary Productivity in Fresh Waters, 2nd edn.

[91] Andersen T, Hessen DO (1991). Carbon, nitrogen, and phosphorus content of freshwater zooplankton.. Limnol Oceanogr.

[92] Canter HM (1950). Fungal parasites of the phytoplankton .I. Studies on British chytrids X.. Ann Bot.

[93] Canter HM, Lund JWG (1951). Studies on plankton parasites. III. Examples of the 399 interaction between parasitism and other factors determining the growth of diatoms.. Ann Bot.

[94] Bush AO, Lafferty KD, Lotz JM, Shostak AW (1997). Parasitology meets ecology on its own terms: Margolis et al. revisited.. J Parasit.

[95] Not F, Simon N, BIegala IC, Vaulot D (2002). Application of fluorescent in situ hybridization coupled with tyramide signal amplification (FISH-TSA) to assess eukaryotic picoplankton composition.. Aquat Microb Ecol.

[96] Carrias JF, Amblard C, Quiblier-Lloberas C, Bourdier G (1998). Seasonal dynamics of free and attached heterotrophic nanoflagellates in an oligomesotrophic lake.. Freshwater Biol.

[97] Arnous MB, Courcol N, Carrias JF (2010). The significance of transparent exopolymeric particles in the vertical distribution of bacteria and heterotrophic nanoflagellates in Lake Pavin.. Aquat Sci.

[98] Devaux J (1980). Contribution à l'étude limnologique du lac Pavin (France) I: facteurs abiotiques et phytoplancton.. Hydrobiologia.

[99] Bettarel Y, Sime-Ngando T, Amblard C, Carrias JF, Sargos D (2002). The functional importance of bacteriophages in the microbial loop of an oligomesotrophic lake over a diel cycle.. Ann Limnol.

[100] Bettarel Y, Amblard C, Sime-Ngando T, Carrias JF, Sargos D (2003). Viral Lysis, Flagellate Grazing Potential, and Bacterial Production in Lake Pavin.. Microbial Ecol.

[101] Steemann-Nielsen E (1952). The use of radio-active carbon (1 4C) for measuring organic production in the sea.. J Cons Explor Mer.

[102] Petit M, Servais P, Lavandier P (1999). Bacterial production measured by leucine and thymidine incorporation rates in French lakes.. Freshwater Biol.

[103] Simon M, Azam F (1989). Protein content and protein synthesis rates of planktonic marine bacteria.. Mar Ecol Prog Ser.

[104] Weinbauer MG, Winter C, Höfle MG (2002). Reconsidering transmission electron microscopy based estimates of viral infection of bacterioplankton using conversion factors derived from natural communities.. Aquat Microb Ecol.

[105] Suberkropp KF, Cantino EC (1973). Utilization of endogenous reserves by swimming zoospores of *Blastocladiella emersonii*.. Arch Microbiol.

[106] Kay JJ, Graham LA, Ulanowicz RE, Wulff F, Field JG, Mann KH (1989). A detailed guide to network analysis.. Network Analysis in Marine Ecology: Methods and Applications.

[107] Ulanowicz RE, Wulff F, Cole J, Lovett G, Findlay S (1991). Comparing ecosystem structures: the Chesapeake Bay and the Baltic Sea.. Comparative analysis of ecosystems, pattern, mechanism, and theories.

[108] Baird D, Luczkovich J, Christian RR (1998). Assessment of spatial and temporal variability in system attributes of the St. Marks National Wildlife Refuge, Apalachee Bay, Florida.. Estuar Coast Shelf S.

[109] Ulanowicz RE, Norden JS (1990). Symmetrical overhead in flow networks.. Int J Syst Sci.

[110] Baird D, Heymans JJ (1996). Assessment of ecosystem changes in response to freshwater inflow of the Kromme River estuary, St. Francis Bay, South Africa: a network analysis approach.. Water S A (Pretoria).

[111] Rutledge RW, Bacore BL, Mulholland RJ (1976). Ecological stability: an information theory standpoint.. J Theor Biol.

[112] Baird D, Asmus H, Asmus R (2004). Energy flow of a boreal intertidal ecosystem, the Sylt-Rømø Bight.. Mar Ecol Prog Ser.

[113] De Angelis DL (1980). Energy Flow, Nutrient Cycling, and Ecosystem Resilience.. Ecology.

[114] Pimm SL (1991). The Balance of Nature?: Ecological Issues in the Conservation of Species and Communities..

[115] Finn JT (1976). Measures of ecosystem structure and function derived from analysis of flows.. J Theor Biol.

[116] Lindeman RL (1942). The trophic dynamic aspect of ecology.. Ecology.

[117] Ulanowicz RE, Kemp WM (1979). Toward a canonical trophic aggregation.. Am Nat.

[118] Ulanowicz RE, Kay JJ (1991). A package for the analysis of ecosystem flow networks.. Env Software.

[119] Tukey JW (1977). Exploratory Data Analysis.

