# Supplementary material for: Functional Effects of Parasites on Food Web Properties during the Spring Diatom Bloom in Lake Pavin: A Linear Inverse Modeling Analysis
Source: PLoS One. 2011 Aug 22;6(8):e23273. doi: 10.1371/journal.pone.0023273 (PMC3161741; doi:10.1371/journal.pone.0023273)
Supplement: Table S1 — Constraints used on different planktonic food web processes. (DOC) [file pone.0023273.s001.doc]

**Table S1.**

Constraints used on different planktonic food web processes.

| Process | | Bound | Description | Equation | Reference |
| --- | --- | --- | --- | --- | --- |
| Gross primary production | ph3 | Upper and lower | GPP of ph3 is comprised between 60% and 85% of total GPP | 60% GPP < GPP-ph3 < 85% GPP | This study |
|  | ph2 | Upper and lower | GPP of ph2 is comprised between 2% and 10% of total GPP | 2% GPP < GPP-ph2 < 10% GPP |
|  | ph1 | Upper and lower | GPP of ph1 is comprised between 5% and 20% of total GPP | 5% GPP < GPP-ph1 < 20% GPP |
| Respiration | ph1 ph2 | Upper and lower | ph2 and ph1 respiration is comprised between 5% and 30% of their GPP | 5% GPP < R < 30% GPP | [S1.1] |
|  | ph3 | Upper and lower | ph3 respiration is comprised between 5% and 40% of their GPP | 5% GPP < R < 40% GPP | [S1.1] |
|  | bac | Lower | Bacteria respiration is at least 20% of their total uptake of doc | 20% U_DOC_ < R | [S1.2] |
|  | hnf, mic mes | Lower | Zooplankton respiration is at least 20% of their total ingestion and doesn't exceed their maximum specific respiration | 20% ΣIng < R | [S1.3] |
|  | Chytrids | Upper | Sporangia and zoospores respiration doesn't exceed 20% of their carbon input | R < 20% C input | This study |
| Doc production | ph1, ph2 ph3 | Upper and lower | Phytoplankton doc exudation is comprised between 10% and 55% of the net primary production (NPP) | 10% NPP < E < 55% NPP | [S1.3] |
|  | hnf, mic mes | Upper and lower | Zooplankton exudation of doc is at least 10% of their total ingestion and doesn't exceed their respiration | 10% ΣIng < E < R | [S1.1;S1.4] |
| Growth efficiency | hnf, mic mes | Upper and lower | The growth efficiency is no more than 50% of the total ingestion (Ing) and is at least 25% of it | 25% ΣIng < Ing -(R + E +Det) < 50% ΣIng | [S1.5] |
|  | bac | Upper and lower | Growth efficiency of bacteria is comprised between 25 % and 50% | 0.5ΣIng < R < 0.75ΣIng | [S1.6] |
| Assimilation efficiency | hnf, mic mes | Upper and lower | Assimilation efficiency of zooplanctonic compartments is comprised between 50 % and 90% of their ingestion | 50% ΣIng < Ing -Det < 90% ΣIng | [S1.5] |
| Grazing of ph3 by mes | | Upper and lower | ph3 grazing by mes is comprised between 3% and 7% of its net primary production | 3% NPP-ph3< Ing _ph3-mes_ < 7% NPP-ph3 | [S1.7] |
| Predation on mic by mes | | Upper | 80% of total ingestion of mesozooplankton | Ing _mic-mes_ < 0.8 ΣIng _mes_ | [S1.5] |
| Preferential ingestion of mes | bac | Upper and lower | bacteria consumption by mes is comprised between 10 and 15% of mes total ingestion | 10%ΣIng mes <Ing bac-mes <15% ΣIng mes | This study |
|  | ph2 | Upper and lower | ph2 grazing by mes is comprised between 10 and 15% of mes total ingestion | 10%ΣIng mes <Ing ph2-mes <15% ΣIng mes |
|  | hnf zsp | Upper and lower | The sum of hnf and zsp consumption by mes is comprised between 15 and 25% of mes total ingestion | 15% ΣIng mes<Ing hnf+zsp-mes < 25% ΣIng mes |
|  | mic | Upper and lower | predation of mes on mic is comprised between 40 and 60% of mes total ingestion | 40%ΣIng mes <Ing mic-mes <60% ΣIng mes |
| Preferential ingestion of mic | bac ph1 | Upper and lower | The sum of bac and ph1 consumption by mic is comprised between 10 and 15% of mic total ingestion | 10%ΣIng mic <Ing bac+ph1-mic <15% ΣIng mic |
|  | ph2 | Upper and lower | ph2 grazing by mic is comprised between 20 and 30% of mic total ingestion | 20%ΣIng mic <Ing ph2-mic <30% ΣIng mic |
|  | hnf zsp | Upper and lower | The sum of hnf and zsp consumption by mic is comprised between 40 and 60% of mic total ingestion | 40%ΣIng mic <Ing hnf+zsp-mic <60% ΣIng mic |
| Preferential ingestion of hnf | bac | Lower | bac consumption by hnf is at least 60% of hnf total ingestion | 60% ΣIng hnf < Ing bac-hnf | This study modified from [S1.8] |
|  | ph1 | Lower | ph1 consumption by hnf is at least 20% of hnf total ingestion | 20% ΣIng hnf < Ing ph1-hnf |
| Detritus production | hnf | Upper | hnf contribution to det carbon input doesn't exceed 20% of its total ingestion | hnf-det < 20% ΣIng _hnf_ | [S1.9] |
|  | mes | Upper | mes contribution to det carbon input doesn't exceed 20% of its total ingestion | mes-det < 20% ΣIng_mes_ |
|  | bac | Upper and lower | Between 1.2% et 5.6% of bacterial production (BP) will contribute to the det carbon input (Attached bacteria) | 1.2% BP < Bac -Det < 5.6% BP | [S1.10] |
|  | ph3 | Upper and lower | Microphytoplankton det production is comprised between 16% and 95% of total det production | 16% Σ Det < ph3-det < 95% Σ Det | [S1.11] |
|  | Chytrids | Upper | Det production by sporangia exceed 5% of its carbon input | Det _spg_ < 5% GPP3-spg | [S1.12] |
| Detritus consumption by mes | | Upper | Mes consumption of detrital is no more than 40% of detritus production | Ing _det-mes_ < 40% Σ Det | This study |
| Detritus dissolution | | Upper | The upper bound of det dissolution is 10% of net particular production | 10% NPP < Diss | [S1.13] |
| Zoospores ingestion | | Lower | Zoospora ingestion by mic is at least twice its ingestion by mes | Ing _zsp-mic_ > 2 Ing _zsp-mes_ | This study |
| Carbon transfer from microphytoplankton to infectious sporangia | | Lower | The lower bound of carbon transfered to sporangia after infections of ph3 cells is 8% of net particular production | gpp-ph3 TO spg > 8% NPP-ph3 | This study modified from [S1.14] |
| Carbon transfert from sporangia to zoospores | | Lower | The lower bound of carbon transfered from sporangia to zoospora is at least the carbon biomass of zoospores compartment | spg TO zsp > Biom zsp | This study |
| Sinking | ph3 | Lower | ph3 sinking is at least 28% of total carbon sinking | ph3-los > 28% Σlos | [S1.15] |
|  |  |  | Sedimentation of ph3 exceed 0.2 mgC m^-2^ d^-1^ | ph3-los > 0.2 | [S1.16] |
|  | mes | Upper and lower | Sedimentation of mes range between 45% and 65% of total sedimentation | 45% Σloss <mes-loss < 65% Σloss | [S1.5] |

**References**

[S1.1] Vézina AF, Platt T (1988) Food web dynamics in the ocean. I. Best-estimates of flow networks using inverse methods. Mar Ecol Prog Ser 42: 269-287.

[S1.2] Vézina AF, Savenkoff C (1999) Inverse modeling of carbon and nitrogen flows in the pelagic food web of the Northeast Subarctic Pacific. Deep-Sea Res PT II 46: 2909-2939.

[S1.3] Breed GA, Jackson GA., Richardson TL (2004) Sedimentation, carbon export, and food web structure in the Mississipi River plume described by inverse analysis. Mar Ecol Prog Ser 278: 35-51.

[S1.4] Vézina AF, Pace ML (1994) An inverse model analysis of planktonic food webs in experimental lakes. Can J Fish Aquat Sci 51: 2034-2044.

[S1.5] Vézina AF, Savenkoff C, Roy S, Klein B, Rivkin R et al. (2000) Export of biogenic carbon and structure and dynamics of the pelagic food web in the Gulf of St. Lawrence. Part 1 Seasonal variations. Deep Sea Res PT II 47: 585-607.

[S1.6] Vézina AF, Pahlow M (2003) Reconstruction of ecosystem flows using inverse methods: how well do they work? J Marine Syst 40-41: 55-77.

[S1.7] Quiblier-Loberas C, Bourdier G, Amblard C, Pepin D (1996) Impact of grazing on phytoplankton in Lake Pavin (France) : Contribution of different zooplankton groups. J Plankton Res 18 (3): 305-322.

[S1.8] Bettarel Y, Amblard C, Sime-Ngando T, Carrias JF, Sargos D et al. (2003) Viral Lysis, Flagellate Grazing Potential, and Bacterial Production in Lake Pavin. Microbial Ecol 45: 119–127.

[S1.9] Carrias JF, Amblard C, Quiblier-Lloberas C, Bourdier G (1998) Seasonal dynamics of free and attached heterotrophic nanoflagellates in an oligomesotrophic lake. Freshwater Biol 39: 91–101.

[S1.10] Lemarchand C, Jardillier L, Carrias JF, Richardot M, Debroas D et al. (2006) Community composition and activity of prokaryotes associated to detrital particles in two contrasting lake ecosystems. FEMS Microbial Ecol 57: 442-451.

[S1.11] Arnous MB, Courcol N, Carrias JF (2010) The significance of transparent exopolymeric particles in the vertical distribution of bacteria and heterotrophic nanoflagellates in Lake Pavin. Aquat Sci 72: 245–253.

[S1.12] Niquil N, Kagami M, Urabe J, Christaki U, Viscogliosi E et al. (2011) Potential role of fungi in plankton food web functioning and stability : a simulation analysis based on Lake Biwa inverse model. Hydrobiologia 659: 65-79.

[S1.13] Pace ML, Glasser JE, Pomeroy LR (1984) A simulation analysis of continental shelf food webs. Mar Biol 82: 47–63.

[S1.14] Kagami M, Gurung TB, Yoshida T, Urabe J (2006) To sink or to be lysed? Contrasting fate of two large phytoplankton species in Lake Biwa. Limnol Oceanogr 51(6): 2775-2786.

[S1.15] Kagami M (2002) Population dynamics and functions of large phytoplankton in Lake Biwa. PhD thesis, Center for Ecological Research Kyoto University.

[S1.16] Carrias JF, Amblard C, Quiblier-Lloberas C, Bourdier G (1998) Seasonal dynamics of free and attached heterotrophic nanoflagellates in an oligomesotrophic lake. Freshwater Biol 39: 91–101.
